# COMPARISON OF SLEEP PARAMETERS FROM WRIST-WORN ACCELEROMETERS IN OLDER ADULTS

**DOI:** 10.1093/geroni/igac059.1307

**Published:** 2022-12-20

**Authors:** Fangyu Liu, Jennifer Schrack, Sarah Wanigatunga, Jill Rabinowitz, Linchen He, Amal Wanigatunga, Eleanor Simonsick, Adam Spira

**Affiliations:** Johns Hopkins Bloomberg School of Public Health, Baltimore, Maryland, United States; Johns Hopkins Bloomberg School of Public Health, Baltimore, Maryland, United States; Johns Hopkins, University, Baltimore, Maryland, United States; Johns Hopkins Bloomberg School of Public Health, Baltimore, Maryland, United States; Johns Hopkins Bloomberg School of Public Health, Baltimore, Maryland, United States; Johns Hopkins Bloomberg School of Public Health, Baltimore, Maryland, United States; National Institute on Aging, Baltimore, Maryland, United States; Department of Mental Health, Johns Hopkins Bloomberg School of Public Health, Baltimore, Maryland, United States

## Abstract

Researchers often prefer different accelerometers for measurement of sleep and physical activity (PA) in older adults, but wearing multiple accelerometers increases costs and participant burden. We tested the hypothesis that the Actiwatch-2, commonly used to measure sleep, and the ActiGraph GT9X Link, commonly used to measure PA, yield comparable sleep parameters among 331 participants (aged 71+/-14 years, 50.5% women) who wore them simultaneously for 7+/-1.6 nights in the BLSA. Compared to the Actiwatch-2, the ActiGraph estimated 50% greater wake after sleep onset, 89% longer wake bout length, 18% lower sleep fragmentation index (SFI), 5% lower total sleep time (TST) and 5% higher sleep efficiency. Yet, despite yielding different absolute values of TST and SFI, the devices generated similar between-person (relative) differences in TST and SFI. These devices may only be interchangeable for measurement of some relative sleep parameters in older adults. Studies with both devices and gold-standard polysomnography are needed.

